# Engineering of an Artificial Light-Modulated Potassium Channel

**DOI:** 10.1371/journal.pone.0043766

**Published:** 2012-08-22

**Authors:** Lydia N. Caro, Christophe J. Moreau, Argel Estrada-Mondragón, Oliver P. Ernst, Michel Vivaudou

**Affiliations:** 1 CNRS, Institut de Biologie Structurale, Grenoble, France; 2 CEA, Institut de Biologie Structurale, LabEx ICST, Grenoble, France; 3 Université Grenoble I, Institut de Biologie Structurale, Grenoble, France; 4 Department of Biochemistry, University of Toronto, Toronto, Ontario, Canada; 5 Department of Molecular Genetics, University of Toronto, Toronto, Ontario, Canada; Dalhousie University, Canada

## Abstract

Ion Channel-Coupled Receptors (ICCRs) are artificial receptor-channel fusion proteins designed to couple ligand binding to channel gating. We previously validated the ICCR concept with various G protein-coupled receptors (GPCRs) fused with the inward rectifying potassium channel Kir6.2. Here we characterize a novel ICCR, consisting of the light activated GPCR, opsin/rhodopsin, fused with Kir6.2. To validate our two-electrode voltage clamp (TEVC) assay for activation of the GPCR, we first co-expressed the apoprotein opsin and the G protein-activated potassium channel Kir3.1_F137S_ (Kir3.1*) in *Xenopus* oocytes. Opsin can be converted to rhodopsin by incubation with 11-*cis* retinal and activated by light-induced retinal *cis→trans* isomerization. Alternatively opsin can be activated by incubation of oocytes with all-*trans*-retinal. We found that illumination of 11-*cis*-retinal-incubated oocytes co-expressing opsin and Kir3.1* caused an immediate and long-lasting channel opening. In the absence of 11-*cis* retinal, all-*trans*-retinal also opened the channel persistently, although with slower kinetics. We then used the oocyte/TEVC system to test fusion proteins between opsin/rhodopsin and Kir6.2. We demonstrate that a construct with a C-terminally truncated rhodopsin responds to light stimulus independent of G protein. By extending the concept of ICCRs to the light-activatable GPCR rhodopsin we broaden the potential applications of this set of tools.

## Introduction

Rhodopsin represents one of the most studied G Protein-Coupled Receptors (GPCRs) and has long been considered as a model for GPCRs. Its crystallographic structure has been solved in different conformations providing important information into the molecular functioning of this receptor [1]–[9]. Expressed in discs of rod cells in the retina, rhodopsin is in charge of dim light vision. Rhodopsin results from the covalent binding of 11-*cis* retinal, a photoisomerizable chromophore derived from vitamin A, to Lys296 of the apoprotein opsin. Upon photon absorption, 11-*cis* retinal is isomerized to the all-*trans* form. As a result, the receptor undergoes conformational changes and a succession of different states yields batho-, lumi-, and metarhodopsin I and II. In metarhodopsin, hydrolysis of the Schiff base between retinal and Lys296 occurs. Among these forms, metarhodopsin II is able to activate a particular heterotrimeric G protein, transducin. Transducin belongs to the pertussis toxin-sensitive G protein family (G_i/o_) and is exclusively expressed in the retina.

G protein-activated inwardly rectifying K^+^ (GIRK or Kir3×) channels [10] regulate cell excitability by modulating membrane resting K^+^ permeability. There are four main types of GIRK channels: Kir3.1, Kir3.2, Kir3.3 and Kir3.4. These are only expressed as heterotetramers in different organs or tissues, such as brain or heart. Nevertheless, it has been reported that point mutations in Kir3.1 (F137S) and in Kir3.4 (S143T) enable surface expression of functional homotetramers [11], [12]. The G protein-sensing capacity of Kir3× channels has already been exploited to report the activity of various GPCRs in *Xenopus* oocytes, including β_2_-adrenergic [13], cannabinoid [14], chemokine [15], dopaminergic [16], histamine [17], and metabotropic glutamate receptors [18].

To our knowledge, no attempt has been made yet to co-express and analyze opsin and GIRK channels in *Xenopus* oocytes. However, two reports of rhodopsin expression and light-induced currents in *Xenopus* oocytes have been published [19], [20]. In both cases, after incubation of rhodopsin-expressing oocytes with 11-*cis* retinal, light application led to the transient appearance of current spikes due to an endogenous Cl^-^ conductance activated through a Ca^2+^-dependent signaling cascade.

In the initial part of this work, we demonstrate G protein-mediated coupling between opsin/rhodopsin and the channel Kir3.1_F137S_ (Kir3.1*) in *Xenopus* oocytes, yielding signal amplitudes two orders of magnitude larger than those measured in other opsin studies in oocytes. We then employed similar assays to characterize the artificial light-gated potassium channel, Ops-Kir6.2, a fusion protein between opsin and Kir6.2, that was built using the blueprints of previous Ion Channel-Coupled Receptors [ICCRs] [21], [22]. Our data established direct coupling between receptor and channel. As shown previously, the surface expression of these constructs was too low for functional characterization but could be enhanced significantly by co-expression with TMD0, the first transmembrane domain of the sulfonylurea receptor (SUR) [22]. Efficient receptor-channel coupling required deletion of most of opsin’s cytosolic C-terminus.

A preliminary report of this work has been presented in abstract form [23].

## Results

### Opsin Activation Potentiates Activity of a Co-expressed G Protein-gated Channel KIR3.1*

**Figure 1:**
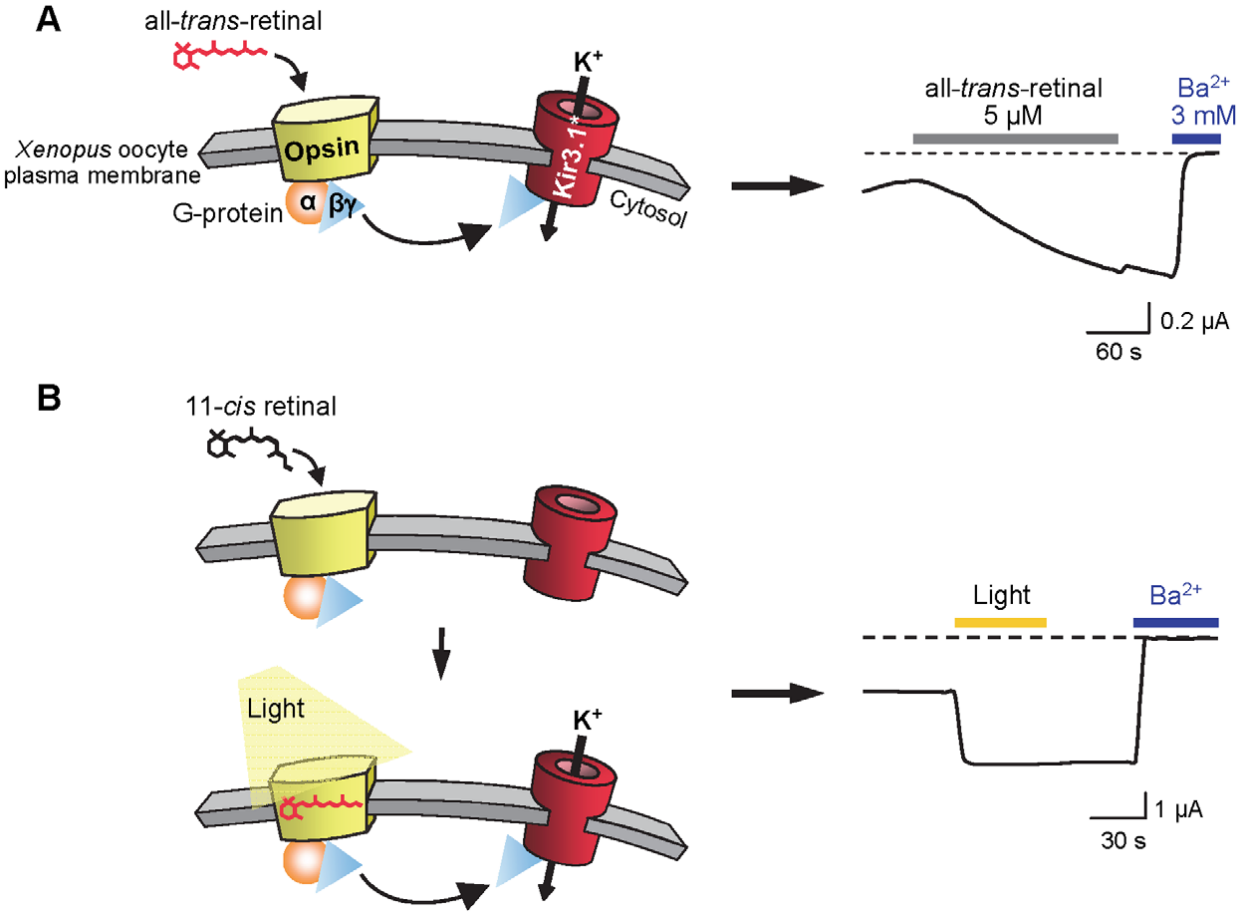
Use of a G protein-activated channel as a reporter of opsin activation. (**A**) Activation by all-*trans*-retinal. Direct application of all-*trans*-retinal to *Xenopus* oocytes expressing opsin and Kir3.1* (Kir3.1_F137S_) leads to Kir3.1* activation via G proteins (Gαßγ). (**B**) Activation by light. Oocytes were pre-incubated with 20 µM 11-*cis* retinal for >30 min in the dark to form rhodopsin. Visible light exposure isomerizes 11-*cis* retinal to all-*trans*-retinal (red), thus activating rhodopsin and, in turn, G proteins which release Gßγ to open Kir3.1*. The traces are representative TEVC recordings from *Xenopus* oocytes expressing opsin and Kir3.1*. Current amplitude was recorded at −50 mV. Dashed line indicates the Ba^2+^-sensitive current baseline.

**Figure 2:**
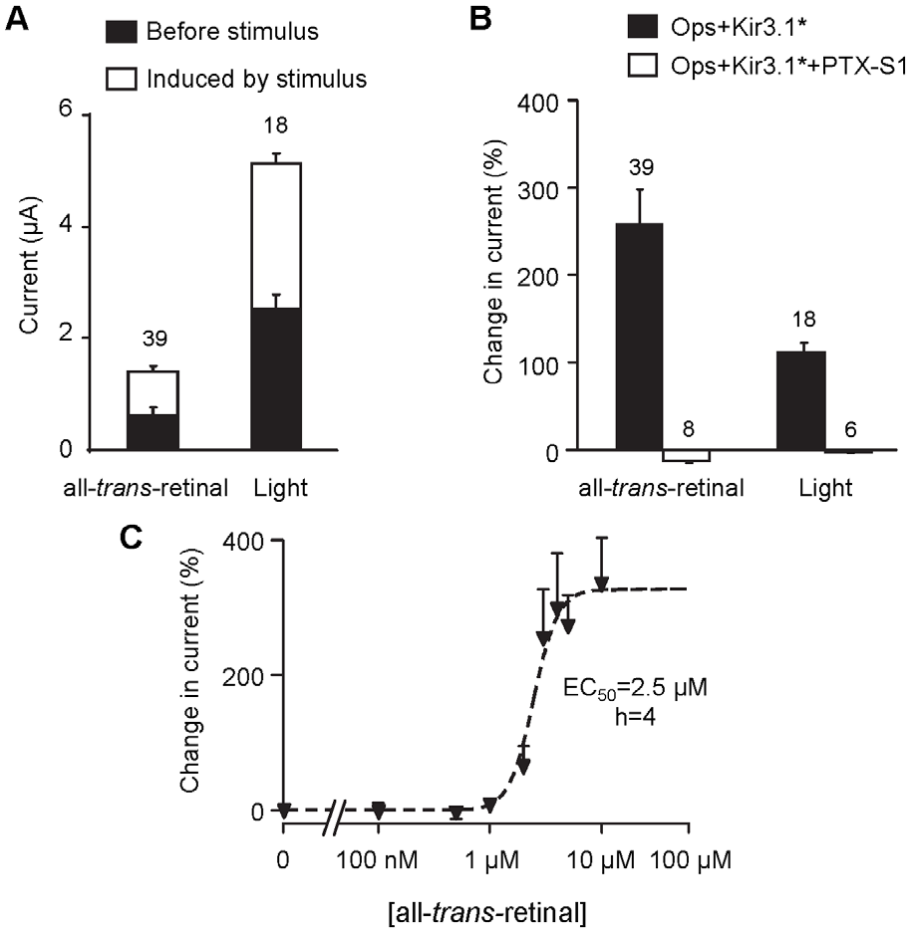
PTX-sensitive activation of Kir3.1* by opsin and all-*trans*-retinal, or light-activated rhodopsin. (**A**) *Xenopus* oocytes were injected with opsin and Kir3.1* mRNAs. Current amplitude was recorded at −50 mV. Black bars represent the average current measured prior to all-*trans*-retinal application or in the dark (after 11-*cis* retinal incubation) in the case of light activation. White bars represent the average current induced by 5 µM all-*trans*-retinal or light stimulation, respectively. Numbers above bars denote the number of oocytes tested. (**B**) Percent change in current induced by application of either 5 µM all-*trans*-retinal or light (after 11-*cis* retinal incubation) in control (black bars) and in the presence of co-expressed catalytic subunit S1 of pertussis toxin (PTX-S1) (white bars). Changes in current were computed for each oocyte and then averaged (The resulting average changes are different from the changes in average current represented in panel A). (**C**) Concentration-dependent response to all-*trans*-retinal. Average data computed as in panel B. Line corresponds to Hill equation fit with h = 4 and EC_50_ = 2.5 µM. Each point represents the average of 7 to 40 measurements.

To demonstrate that Kir3.1* can serve as a reporter for the activation of opsin in *Xenopus* oocytes, we examined its response to the opsin agonist all-*trans*-retinal and to photoactivation of 11-*cis* retinal-reconstituted rhodopsin. All-*trans*-retinal was tested directly on oocytes expressing opsin and Kir3.1*. Application of all-*trans*-retinal at 5 µM led to a 260±40% increase in Ba^2+^-sensitive current activation (Fig. 1A and Fig. 2). This increase developed slowly and did not reach a maximum even several minutes after the application of the agonist. Because oocytes impaled with microelectrodes tend to develop large leak currents over time, measurements were not continued for more than 5 minutes. Although this duration was insufficient to reach a plateau as evidenced in Fig. 1A, it gave a good estimate of maximal activation. After a wash step to remove all-*trans*-retinal, the activation did not reverse significantly within the time frame of our experiments, i.e., for up to 4 minutes. However, the all-*trans*-retinal-induced current declined when hydroxylamine, which is known to inactivate the receptor due to retinal oxime formation, was added to the oocytes (Fig. S1). To assess possible non-specific interactions of all-*trans*-retinal with Kir3.1* or with endogenous oocyte conductances, we examined the response of oocytes expressing Kir3.1* alone (Fig. S2). These experiments confirmed that all-*trans*-retinal elicits no current modulation in the absence of opsin.

Activation by light requires the presence of the light-sensitive chromophore 11-*cis* retinal. Because of the difficulty of conducting experiments in the dark, oocytes were first screened with TEVC to eliminate poorly-expressing oocytes, as evidenced by the absence of inward rectifying currents. Selected oocytes were incubated in the dark with 20 µM 11-*cis* retinal to allow binding of 11-*cis* retinal to opsin to form rhodopsin. After at least 30 minutes, TEVC tests with white light were performed. Illumination caused an immediate increase in current of 111±11% (Fig. 1B and Fig. 2A&B). As with all-*trans*-retinal, this effect did not appreciably reverse after light was switched off (measured for up to 50 s). The activation evoked by all-*trans*-retinal can be compared qualitatively but not quantitatively with activation evoked by 11-*cis* retinal and illumination because of differences in basal currents. Indeed, the oocytes used for the more complex light-stimulation experiments were subjected to a preliminary selection (see above and Methods) and therefore had larger basal currents.

A common feature emerges from these data: in both cases, receptor activation was poorly reversible within the allowed 1–10 minute time frame of our recordings. This could be due to the fact that all-*trans*-retinal (added directly or produced after light-induced isomerization of 11-*cis* retinal) diffuses slowly out of its binding site and maintains the receptor active, and/or that the hydrophobic retinal partitions into the membrane and cannot be washed out effectively. It was recently shown directly in opsin crystals that all-*trans*-retinal can reconstitute the active form of rhodopsin, metarhodopsin II, with the ligand covalently bound in the retinal binding pocket [4]. However, hydroxylamine showed the known behavior that formation of retinal oxime hinders receptor activity (i.e., of light-induced metarhodopsin II or opsin/all-*trans*-retinal). We noticed that the all-*trans*-retinal activation kinetics were very slow. Moreover, it was sometimes difficult to reach maximal activation because of the required recording length and concomitant oocyte death. This slowness could reflect impaired access of all-*trans*-retinal to its binding site. Uptake of all-*trans*-retinal by opsin appears to depend on the presence of an active opsin conformation which is in equilibrium with an inactive conformation [24]. Accordingly, the oocyte membranes may favor the inactive opsin conformation.

Concentration-response experiments with all-*trans*-retinal were performed in order to determine its affinity for opsin. Increasing concentrations of all-*trans*-retinal applied to oocytes elicited a steep concentration-response curve (Fig. 2C). Curve fitting to the experimental data led to a Hill coefficient of 4, which suggests a high cooperativity between the receptors present at the membrane. This might be due to a property of the native membranes of the *Xenopus* oocytes, in which membrane proteins form clusters as shown by atomic force microscopy (AFM) analysis [25]. Additionally, although monomeric rhodoposin is the smallest functional unit of the GPCR [26]–[29], rhodopsin can oligomerize in the rod cell disk membrane [30]–[32] and may naturally tend to form tetramers in *Xenopus* oocytes membranes, thus explaining the Hill coefficient of 4 obtained in presence of all-*trans*-retinal. The presence of such receptor oligomers may have an effect on Schiff base hydrolysis and/or an allosteric or steric effect on retinal release which may explain the sustained activity observed when light stimulation was switched off (Fig. 1B).

### Rhodopsin Interacts with Gi/o Proteins in Xenopus Oocytes

In the retina, rhodopsin signaling is mediated by the heterotrimeric G protein transducin. There is no evidence of the presence of transducin in *Xenopus* oocytes, but it has been shown that rhodopsin can efficiently interact with G_i_ proteins [33], [34]. We assume that recombinant rhodopsin couples to endogenous G_i/o_ proteins in *Xenopus* oocytes to activate Kir3.1*. To confirm this, we co-expressed the receptor with Kir3.1* and the catalytic subunit of pertussis toxin (PTX-S1) [12], [21]. PTX-S1 ADP-ribosylates the α subunit of G proteins from the G_i/o_ family and this prevents coupling of GPCR and G protein [35]. As a result, free βγ subunits are no longer available to regulate Kir3.1* gating [12]. In opsin/Kir3.1*/PTX-S1 co-expression experiments we measured the change in current induced by either the application of all-*trans*-retinal or by illumination (after 11-*cis* retinal incubation). In both cases, PTX-S1 abolished the response elicited by the stimulus (Fig. 2B). We concluded that recombinant rhodopsin signals to Kir3.1* through endogenous G proteins present in oocytes.

### Design of Opsin-based ICCRs and Characterization of Opsin-Kir6.2 Coupling

**Figure 3:**
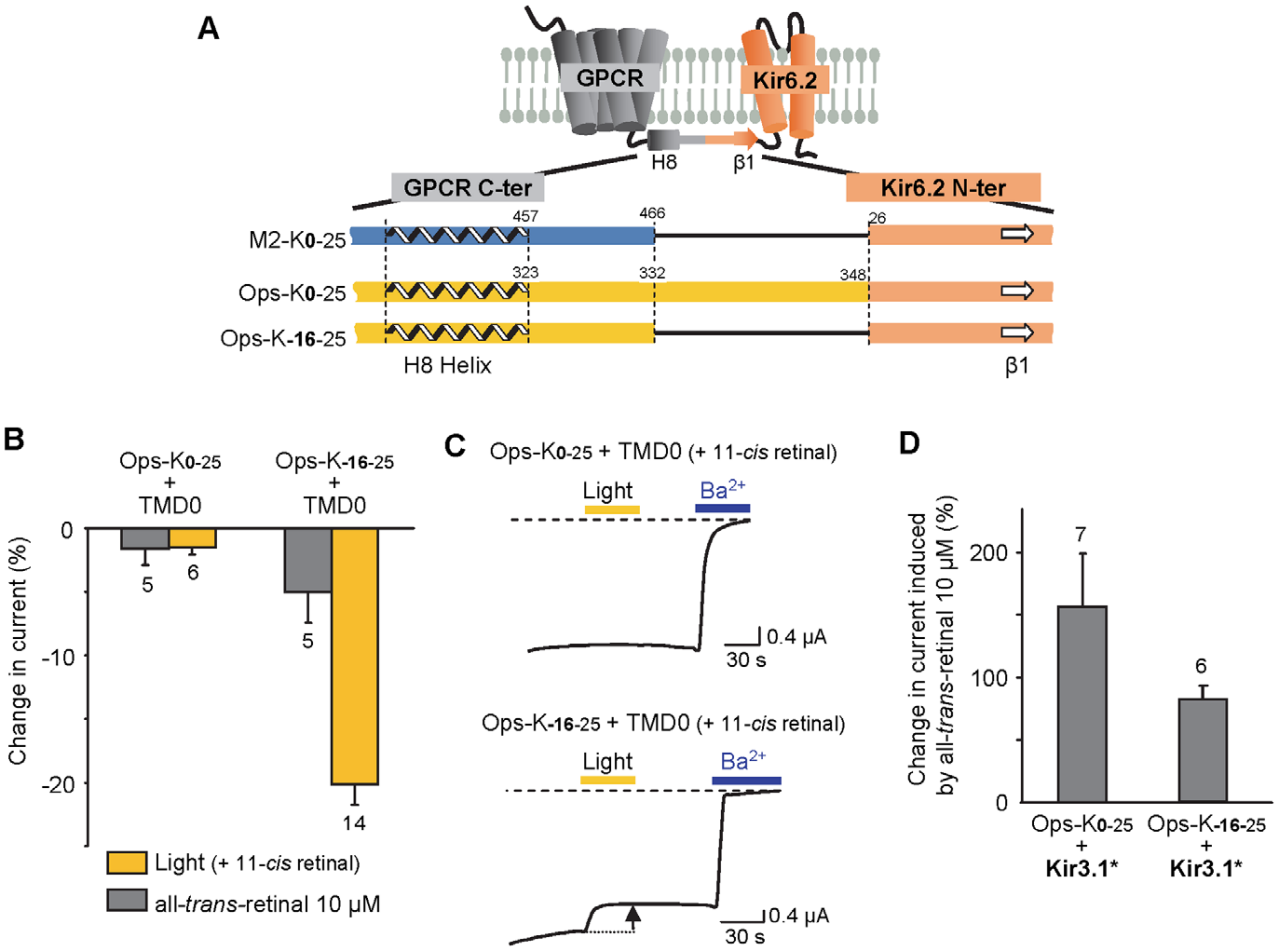
Design strategy of rhodopsin-based ICCRs and functional coupling. (**A**) Sequence alignment of the fusion area between GPCRs and Kir6.2ΔN25. Alignment of GPCR C-terminal sequences were based on the presence of the H8 Helix. (**B**) Percent change in current for each construct in response to 10 µM all-*trans*-retinal or light (after 11-*cis* retinal incubation). Oocytes were co-injected with the specified Ops-Kir6.2 and TMD0, a SUR transmembrane domain. Numbers below bars indicate the number of oocytes tested. The changes induced by all-*trans*-retinal and light were statistically significant for Ops-K_−16–25_ (Student t-test; P<0.04 & P<0.0001, respectively), but not for Ops-K_0–25_ (P>0.4 & P>0.05, respectively). (**C**) Representative TEVC recordings for each construct in the case of photoactivation. Yellow bar represents oocyte illumination. Blue bar corresponds to Ba^2+^ application at 3 mM. Dashed line indicates the Ba^2+^-sensitive current baseline. (**D**) Opsin ability to activate G proteins within the fusion Ops-Kir6.2. Both constructs were co-expressed with Kir3.1* and change in current was measured in response to 10 µM all-*trans*-retinal. All measurements were done at −50 mV. Numbers above bars indicate the number of oocytes tested.

After testing functional expression of opsin in oocytes using Kir3.1* as a reporter, we designed two opsin-based ICCRs following the strategy adopted for the design of β_2_-adrenergic receptor-based ICCRs [22]. The first one uses the full-length receptor (Ops-K_0-25_) and the second, a truncated version of it, with 16 amino acids deleted from the opsin C-terminus (Ops-K_−16−25_). The number of amino acids deleted was determined from the alignment of the C-terminal sequences of the muscarinic M_2_ receptor and opsin (Fig. 3A).

As shown previously with β_2_-adrenergic receptor-based ICCRs [22], sufficient surface expression of the opsin-Kir6.2 constructs could only be achieved by co-expressing TMD0, the N-terminal transmembrane domain of SUR [36] and a key element of the SUR-Kir6.2 interaction [37]. Using this strategy, the constructs were challenged with two types of stimuli: either 10 µM all-*trans*-retinal, or flash illumination after incubation with 20 µM 11-*cis retinal* in the dark. The construct based on the full-length receptor, Ops-K_0–25_, did not respond to either stimulus whereas Ops-K_−16–25_ was light sensitive (Figs. 3B and 3C). Indeed, illumination of oocytes expressing Ops-K_−16–25_+ TMD0 resulted in a 20% decrease in current. All-*trans*-retinal at 10 µM caused a small (<5%) but significant change in current (Fig. 3B). This difference between the two stimuli could be explained by the possibility that there is a significantly lower affinity of exogenous all-*trans*-retinal for Ops-K_−16–25_ compared with opsin due to a putative allosteric effect of Kir6.2 on the opsin conformation. Further, the covalent linkage of retinal in the ligand binding site during photochemical generation of all-*trans*-retinal from its 11-*cis* form obviates the need for the *in situ* generated all-*trans*-retinal to have high affinity. Alternatively, exogenous all-*trans*-retinal and all-*trans*-retinal produced *in situ* in the retinal binding pocket from light isomerization of 11-*cis* retinal could induce different conformational changes.

In order to check the integrity of opsin within the opsin-Kir6.2 fusion protein (i.e., the ability of opsin to activate G proteins), Ops-K_0–25_ and Ops-K_−16–25_ were co-expressed with the reporter channel Kir3.1*. In both cases a large increase in current by 10 µM all-*trans*-retinal was measured, even though that increase remained less important than with opsin alone. The percent increases were 150% for Ops-K_0–25_+ Kir3.1* and ∼80% for Ops-K_−16–25_+ Kir3.1* (Fig. 3D), compared with 351% for opsin + Kir 3.1* (Fig. 2C). However, beyond the qualitative conclusion that opsin remains functional in terms of G protein activation within the fusions, it is difficult to draw any inference from the values because the lesser current increase mediated by the fusions could be due to lower levels of expression of the fused receptor compared to the isolated receptor.

### G proteins and Opsin-Kir6.2 Coupling

**Figure 4:**
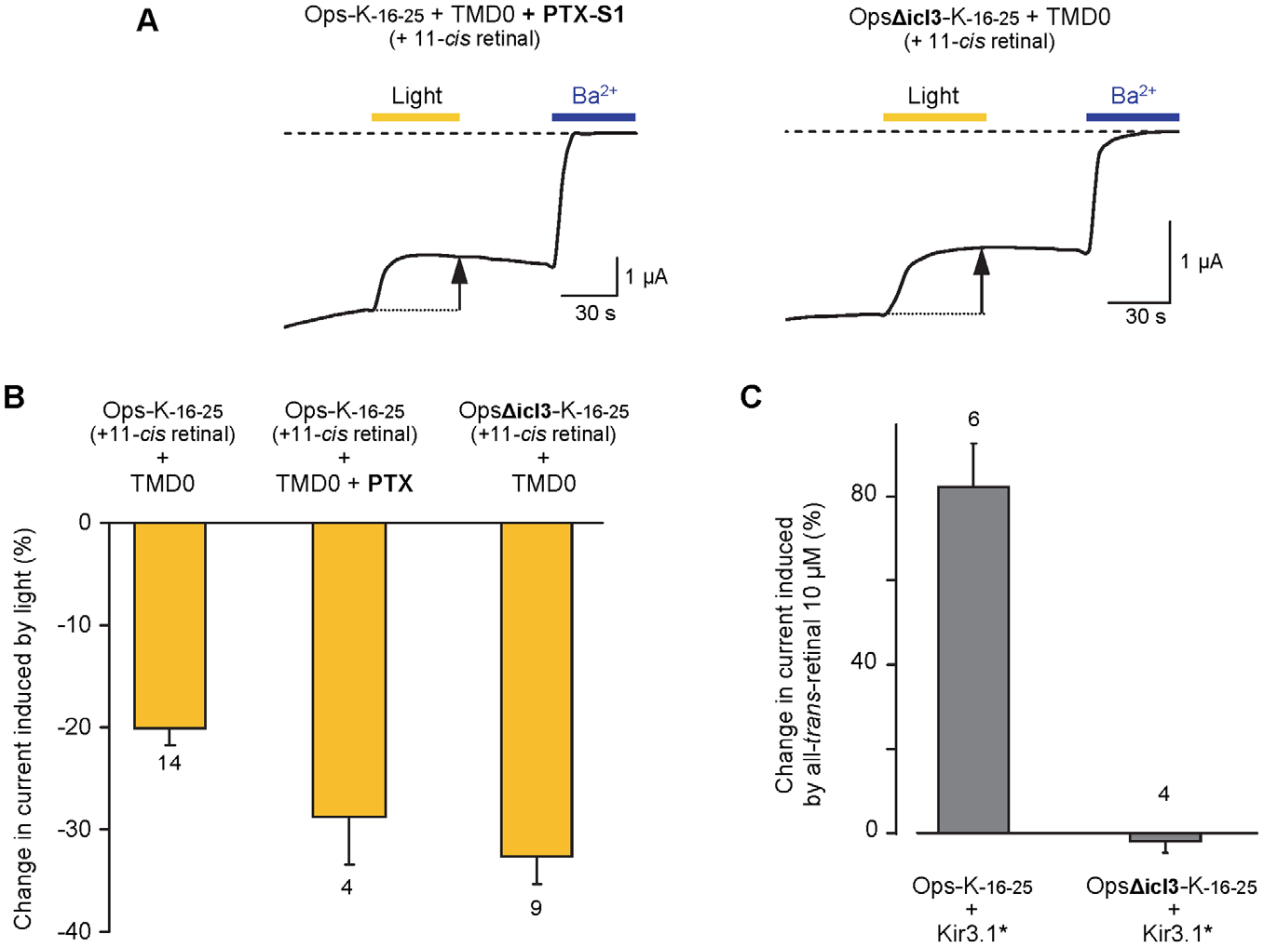
ICCRs report conformational changes of receptors deficient in G protein coupling. (**A**) Representative TEVC traces. Yellow and blue bars correspond to oocyte illumination and Ba^2+^ (3 mM) application, respectively. PTX (Pertussis Toxin) ADP-ribosylates G_αi/o_ proteins, thus preventing G_α_-G_βγ_ dissociation. OpsΔicl3 is a rhodopsin mutant with a deletion in the third intracellular loop (Δ244–249) known for its inability to activate G proteins. Dashed line indicates the Ba^2+^-sensitive current baseline. Measurements were done at −50 mV. (**B**) Percent change in current induced by light after 11-*cis* retinal incubation in the dark. Numbers below bars indicate the number of oocytes tested. The responses of OpsΔicl3-K_-16–25_ and Ops-K_-16–25_+ PTX were not statistically different (Student t-test; P>0.5) while those of OpsΔicl3-K_-16–25_ and Ops-K_-16–25_ were different (P<0.002). (**C**) Oocytes were co-injected with Ops-K_-16–25_+ Kir3.1* or OpsΔicl3-K_−16–25_+ Kir3.1*. Ability of opsin to activate G proteins was determined by the addition of all-*trans*-retinal at 10 µM and measurement of Kir3.1* activation as a percent change in current. Numbers above bars indicate the number of oocytes tested.

We used two approaches to test whether the GPCR and channel couple directly in opsin-based ICCRs or whether the ICCR requires G protein coupling for channel activation. First we co-expressed PTX-S1 with Ops-K_−16–25_+ TMD0. Light-stimulation experiments showed that communication between opsin and Kir6.2 was not impaired in the presence of PTX (Figs. 4A and 4B). Because G proteins modified by PTX become unable to bind to receptors [38], this observation provides evidence that G protein binding to opsin is not required for the ICCR coupling mechanism. These results corroborate our previously published observations with the M2 muscarinic receptor [21]. In a second approach, we tested an opsin mutant with a deletion in intracellular loop 3 (icl3) rendering the GPCR unable to activate G proteins [39], [40]. In the ICCR OpsΔicl3-K_−16–25_, six residues of opsin’s icl3 were deleted (Δ244–249) [40]. The disruption of G protein activation by the Δ244–249 deletion was verified by the failure of OpsΔicl3-K_−16–25_ to trigger activation of Kir3.1* upon all-*trans*-retinal application (Fig. 4C). To test the effect of the deletion on the ICCR, the construct OpsΔicl3-K_-16−25_ was co-expressed with TMD0 and subjected to light exposure after 11-*cis* retinal incubation in the dark (Fig. 4B). This response, a decrease in current of >32%, was not statistically different from the response of Ops-K_−16–25_ measured in the presence of PTX, but somewhat larger than the response of Ops-K_−16–25_ alone. Because we have not seen any significant effect of receptor-activated G-proteins on Kir6.2, either fused to a GPCR (as shown with Ops-K_0–25_ in Fig. 3C) or as an isolated channel [21], these results suggest that activated G proteins might still have some affinity for Ops-K_−16–25_, thereby reducing the amplitude of the ICCR signal. Such a behavior could be related to a previous observation suggesting that upon GTP uptake some G proteins undergo subunit rearrangement instead of dissociation [41], [42].

### Possible Mechanism of Receptor-channel Coupling in ICCRs

**Figure 5:**
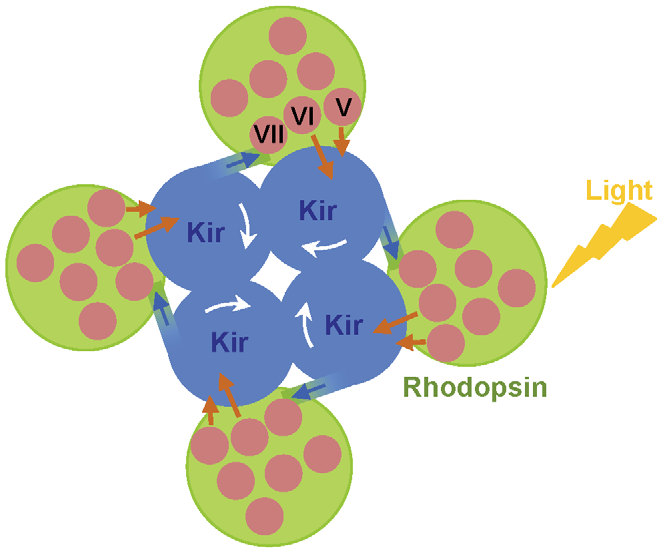
Possible mechanism linking channel gating to ligand binding. This sketch is a view of the opsin ICCR from the cytoplasmic side. The ICCR contains four Kir6.2 subunits (labeled Kir) that associate as a tetramer and four opsin receptors at the periphery. One hypothesis for the observed coupling between light activation of rhodopsin and channel gating could be that closing of the channel results from light-induced motion of receptor-channel linker (blue arrows) and rearrangement of opsin helices TM-V and TM-VI (brown arrows) (see text for details).

The increasing number of GPCR structures solved in their active state [4], [5], [7], [43]–[45] suggests that rigid body rotational outward movement of the kinked transmembrane helix (TM) VI [5], [46] is a common feature of GPCR activation. Further, upon rhodopsin activation, TM-V moves towards TM-VI, and TM-VII with the attached cytoplasmic helix 8 moves slightly towards the helix bundle [47]. With a possible interaction between rhodopsin TM-V/TM-VI and the transmembrane region of Kir6.2, the TM-V/TM-VI rearrangement could push the neighboring Kir6.2 subunit (Fig. 5). Given that the Δ244–249 deletion (icl3) removes the cytoplasmic tip of TM-VI, it appears that the whole transmembrane part (including the extracellular end) of TM-VI is used to transfer the signal to Kir6.2. The rotation of TM-VI and movement of TM-V would trigger a twisting motion of each Kir6.2 subunit and could lead to ion channel closing. The geometric constraints for such a mechanism would also explain why the length of the linker between receptor and channel has to be carefully adjusted [22].

## Discussion

We have shown here that *Xenopus* oocytes are suitable cells to examine the function of opsin using a G protein-activated Kir3.1 channel as reporter or by direct coupling of opsin with Kir6.2.

In response to light or chemical stimuli, rhodopsin or opsin, respectively, is found to couple efficiently to endogenous PTX-sensitive G proteins as evidenced by the activation of the Kir3.1 reporter channel. All-*trans*-retinal was found to behave as an opsin agonist with an affinity of 2.5 µM and an unusually steep concentration dependence that could possibly reflect strong cooperativity between oligomerized receptors at the oocyte cell surface. As a more elaborate tool to directly influence ion channel gating by light, we constructed an ICCR by fusing Kir6.2 with opsin to enable retinal-mediated photosensitivity. As previously published for the β2-adrenergic receptor, coupling between receptor and channel required short deletions of the receptor C-terminus and the channel N-terminus, and sufficient plasma membrane expression required the chaperone help of co-expressed TMD0 domain of the sulfonylurea receptor. Thus we were able to extend the concept of ICCRs to a receptor-channel fusion with covalently bound chromophoric ligand capable of modulating conductance by light.

One posssible use of such an artificial channel, beside its use in GPCR research as a reporter of opsin conformational changes, would be as a means to optically control resting K^+^ permeability in cells that express it. We are aware that such a goal still requires significant improvements. One obstacle, activation of G protein by opsin, was already surmounted by showing that light-sensing by the ICCR could be achieved by a mutant opsin unable to activate G proteins. Other improvements concern the resting activity of the ICCR and the amplitude and sign of the response to light. Ideally, the resting activity should be minimal to avoid disrupting normal cell function in absence of stimulus, and light should increase this activity sufficiently to hyperpolarize the membrane. Presently, the opsin ICCRs are significantly open at rest and are partly closed by light. Further work is therefore required to reduce resting open probability and to reverse the response to light. The former goal could be achieved by introducing mutations in Kir6.2 to reduce its open probability [48] or modify its sensitivity to inhibition by intracellular ATP [49]. The latter goal, inverting the response to produce channel activation upon receptor stimulation, is not out of reach as we already reported that muscarinic [21] and ß-adrenergic ICCRs [22] are activated by agonists. Further, the use of bistable rhodopsin pigments with stable retinal Schiff bases may allow adjustment of the ion flux gradually as a function of the illumination protocol. We are now in the process of deciphering the molecular mechanisms underlying receptor-channel coupling, a necessary step toward the rational design of customized ICCRs.

## Materials and Methods

### Ethics Statement

Animal handling and experiments were conducted in Grenoble, France. They fully conformed to French regulations and were approved by governmental services.

The animal facilities were granted approval number “c 38 185 10 001” on 7 July 2010 by the local government representative (Prefet de l’Isere, Direction Departementale de la Protection des Populations) for the period 7 July 2010 −6 July 2015.

Experiments on *Xenopus laevis* frogs have been authorized (Authorization N°38 08 10 granted to Michel Vivaudou on 22 February 2008, valid from 22 February 2008 to 22 February 2013) by the local veterinary agency (Directeur Départemental des Services Vétérinaires, Ministere de l’Agriculture et de la Peche).

### Molecular Biology

We used a synthetic bovine opsin gene [50] and cDNAs of the mutated channels Kir3.1_F137S_ and Kir3.4S_143T_, designated here Kir3.1* and Kir3.4*, respectively [12], the catalytic subunit of pertussis toxin PTX-S1 [12], [21], mouse Kir6.2 (Genbank D50581) and hamster TMD0(SUR1)-F195 (the kind gift of Dr. Kim Chan) [51]. The Ops-K_0-25_ fusion was obtained by replacing the muscarinic M_2_ receptor gene in M_2_-K_0-25_ cloned in the *Xenopus* oocyte expression vector pGEMHE. Insertion of the opsin gene and deletion of the M_2_ gene was performed using a two-step PCR. In the first PCR reaction, the opsin gene was amplified from its original expression vector [50] using hybrid primers complementary to the opsin sequence 3′ extremities and to the flanking regions of the insertion site in the M_2_-Kir6.2-pGEMHE. The products of this reaction were gel-purified and served as primers for a second PCR with M_2_-K_0–25_ as a template, yielding Ops-K_0-25_-pGEMHE. Alignments of the M_2_, D_2_, and opsin sequences with ClustalX were adjusted manually to position conserved helix H8. The unstructured C-terminal region downstream of H8 was longer in opsin by 16 amino acids compared to M_2_ (Fig. 3). To match the length of the M_2_ receptor in the M2-K_0–25_ fusion construct, the Ops-K_−16–25_ construct with shorter opsin C-terminus was obtained in a single-step PCR using the Ops-K_0-25_ construct as a template and hybrid oligonucleotides flanking the deleted region. After DNA amplification, constructs were linearized and mRNAs synthesized using the T7 mMessage mMachine Kit (AMBION). mRNAs were purified either by standard phenol:chloroform extraction or using the MEGAclear Purification Kit (AMBION), and quantified by agarose-gel electrophoresis and spectrophotometry.

### Electrophysiological Recordings

Oocytes were surgically removed from *Xenopus laevis* and defolliculated by three 30 min-incubations in 2 mg.ml^−1^ type 1A collagenase solution at 19°C. Stage V and VI oocytes were microinjected with 50 nl of RNase-free water containing one or a mixture of the following quantities of RNA: Opsin, 2 ng; Kir3.1*, 2 ng; PTX-S1, 1 ng. Microinjected oocytes were incubated for more than 2 days at 19°C in Barth’s solution (in mM: 1 KCl, 0.82 MgSO4, 88 NaCl, 2.4 NaHCO3, 0.41 CaCl2, 16 Hepes, pH 7.4) supplemented with 100 U.ml^−1^ penicillin, streptomycin and gentamycin. All chemicals were purchased from Sigma-Aldrich. 11-*cis* retinal was prepared from all-*trans*-retinal [52]. Whole-cell currents were recorded with the two-electrode voltage clamp (TEVC) technique using a GeneClamp 500 amplifier (Molecular Devices). Microelectrodes were filled with 3 M KCl and oocytes were bathed in the following solution (TEVC bath, in mM): 91 KCl, 1.8 CaCl2, 1 MgCl2, 5 HEPES, 0.3 niflumic acid (to block endogenous Cl^−^ currents), pH 7.4. The TEVC voltage protocol consisted of 500-ms steps to −50, 0 and +50 mV, during which current was measured, separated by 5 s at a holding potential of 0 mV. The values shown in the figures are those recorded at −50 mV. Basal current was measured while oocytes were in standard bath solution during the first minute of recording.

For photo-stimulation experiments, oocytes were first screened in normal light by TEVC recording to eliminate unhealthy and poorly-expressing oocytes. Positive oocytes, selected on the basis of a large inward-rectifying basal current, were placed in 96-well plates and incubated in a 20 µM 11-*cis* retinal solution (in TEVC bath) for at least 30 min, at room temperature and in dark conditions (i.e., dim red light). Oocytes were then placed in the TEVC chamber and impaled with two electrodes. Basal current was measured in the dark and oocytes were illuminated with white light supplied through a fiber-optic guide by a halogen-tungsten lamp. The intensity was 3.5 mW/cm^2^. After light was switched off, barium (chloride salt; 3 mM) was applied manually, avoiding large modulations of the flux.

Average values are presented as mean ± s.e.m. Statistical significance was assessed with Student’s t-tests for paired (evaluation of effector action) and unpaired (comparison of properties of different constructs).

Non-linear least-square curve-fitting was carried out using a standard Hill equation:
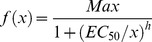
where x is the concentration of a ligand, Max the asymptotical maximal effect, EC_50_ the concentration for half-maximal effect, and h the Hill coefficient. Ba^2+^ (3 mM) was used as a generic potassium-channel blocker to establish the amount of exogenous current. Changes in Ba^2+^-sensitive currents by effectors were usually calculated with respect to a baseline extrapolated from the values measured before and after agonist application. This method emphasizes reversibility and tends to underestimate the true effects. For the concentration-response data, obtained by sequential application of increasing agonist concentrations, changes in current were calculated only with respect to the current before application of agonist.

## Supporting Information

Figure S1 **Hydroxylamine (NH_2_OH) inhibits all-** ***trans*** **-retinal-induced activity of opsin and light-induced activity of rhodopsin.** **(A)** Representative TEVC recording from an oocyte expressing opsin and Kir3.1*. The histogram below shows the average values, computed from experiments in 7 oocytes, of the Ba^2+^-sensitive current at the different steps of the assay. [all-*trans*-retinal] = 5 µM; [NH_2_OH] = 10 mM. **(B)** Same as in panel A except that oocytes were pre-incubated with 11-*cis* retinal and kept in the dark, and that opsin was activated by light. Average values were also computed from 7 experiments. The initial application of hydroxylamine before all-*trans*-retinal application in panel A shows that hydroxylamine affects neither Kir3.1* nor opsin. The reactivation of opsin after wash-out of hydroxylamine is likely due to all-*trans*-retinal accumulated in the oocyte membrane.
(TIFF)

Figure S2 **All-** ***trans*** -retinal does not alter Kir3.1* **activity.** **(A)** Representative TEVC recording from a *Xenopus* oocyte expressing Kir3.1* alone. **(B)** *idem* for an oocyte expressing Kir3.4* alone. Like Kir3.1*, Kir3.4* (Kir3.4 with the mutation S143T) forms homotetrameric K^+^ channels that are activated by G protein ßγ subunits. **(C)** Average changes in Ba^2+^-sensitive whole-cell current evoked by 5 and 10 µM all-*trans*-retinal in oocytes expressing Kir3.1* (Red bars) or co-expressing Kir3.1* and opsin (Black bars). Numbers above bars indicate the number of oocytes tested. All-*trans*-retinal at 5 and 10 µM had no statistically significant effect on Kir3.1* (Student t-test; P>0.4).
(TIFF)
