# Supplementary figures and images for: Engineering of an Artificial Light-Modulated Potassium Channel
Source: PLoS One. 2012 Aug 22;7(8):e43766. doi: 10.1371/journal.pone.0043766 (PMC3425490; doi:10.1371/journal.pone.0043766)

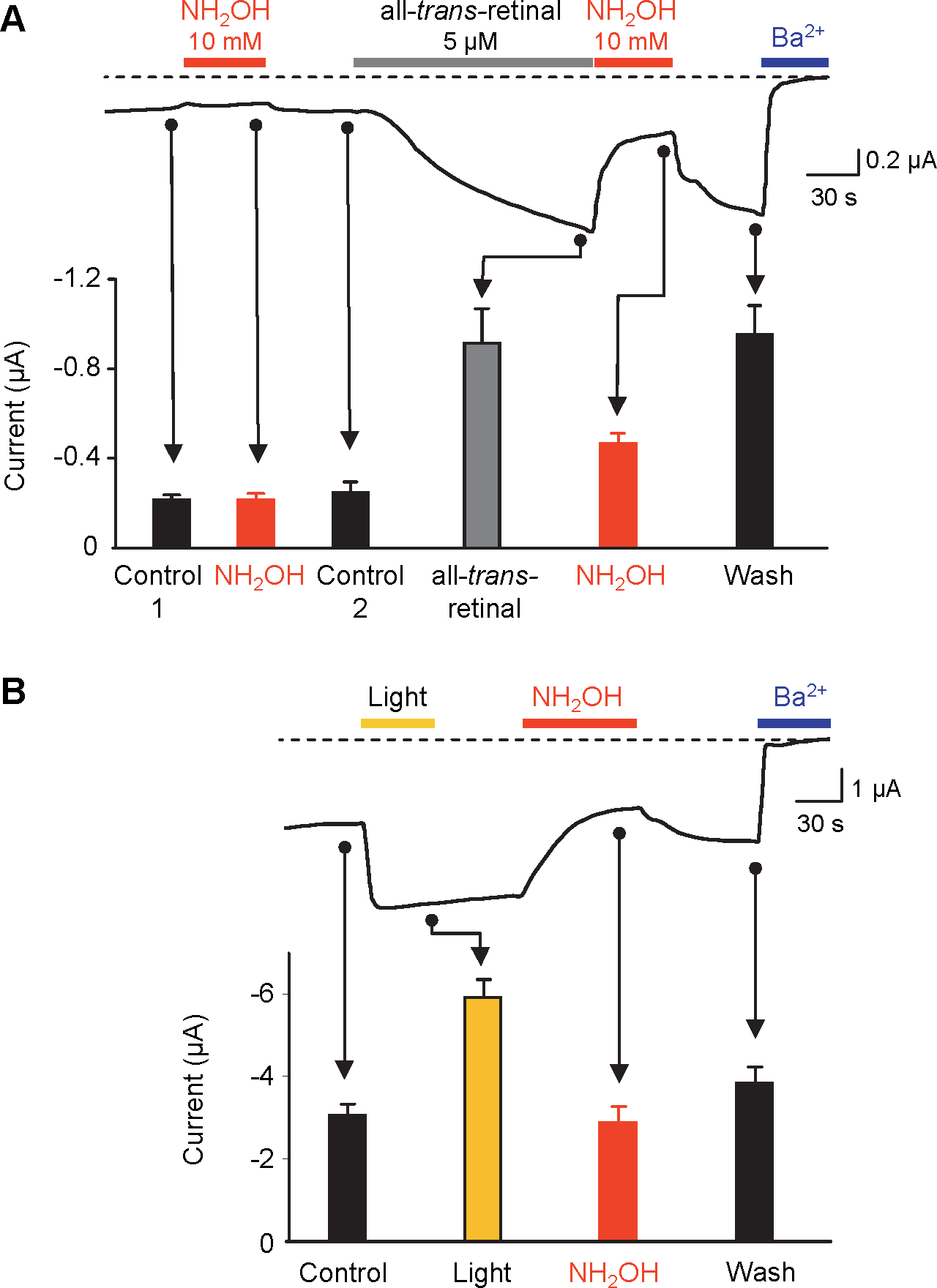

Supplement: Figure S1 — Hydroxylamine (NH2OH) inhibits all- trans -retinal-induced activity of opsin and light-induced activity of rhodopsin. (A) Representative TEVC recording from an oocyte expressing opsin and Kir3.1*. The histogram below shows the average values, computed from experiments in 7 oocytes, of the Ba2+-sensitive current at the different steps of the assay. [all-trans-retinal] = 5 µM; [NH2OH] = 10 mM. (B) Same as in panel A except that oocytes were pre-incubated with 11-cis retinal and kept in the dark, and that opsin was activated by light. Average values were also computed from 7 experiments. The initial application of hydroxylamine before all-trans-retinal application in panel A shows that hydroxylamine affects neither Kir3.1* nor opsin. The reactivation of opsin after wash-out of hydroxylamine is likely due to all-trans-retinal accumulated in the oocyte membrane. (TIFF) [file pone.0043766.s001.tiff]

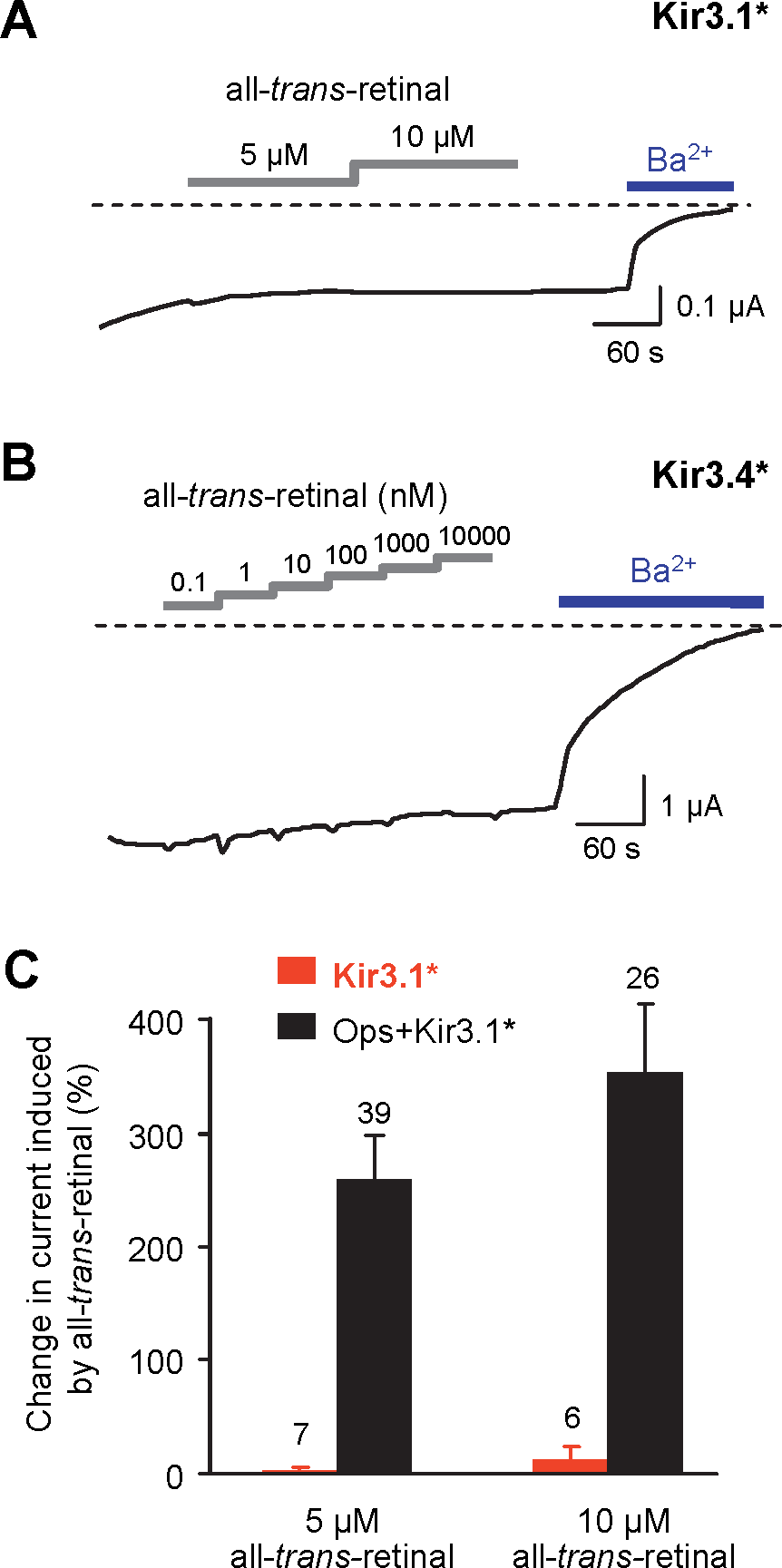

Supplement: Figure S2 — All- trans -retinal does not alter Kir3.1* activity. (A) Representative TEVC recording from a Xenopus oocyte expressing Kir3.1* alone. (B) idem for an oocyte expressing Kir3.4* alone. Like Kir3.1*, Kir3.4* (Kir3.4 with the mutation S143T) forms homotetrameric K+ channels that are activated by G protein ßγ subunits. (C) Average changes in Ba2+-sensitive whole-cell current evoked by 5 and 10 µM all-trans-retinal in oocytes expressing Kir3.1* (Red bars) or co-expressing Kir3.1* and opsin (Black bars). Numbers above bars indicate the number of oocytes tested. All-trans-retinal at 5 and 10 µM had no statistically significant effect on Kir3.1* (Student t-test; P>0.4). (TIFF) [file pone.0043766.s002.tiff]
